# Supplementary material for: Regioselective Claisen–Schmidt Adduct of 2-Undecanone from Houttuynia cordata Thunb as Insecticide/Repellent against Solenopsis invicta and Repositioning Plant Fungicides against Colletotrichum fragariae
Source: Molecules. 2023 Aug 17;28(16):6100. doi: 10.3390/molecules28166100 (PMC10458534; doi:10.3390/molecules28166100)
Supplement: Supplementary file 1 [file molecules-28-06100-s001.zip › molecules-2491275-supplementary.pdf]

Regioselective Claisen–Schmidt Adduct of 2-Undecanone from *Houttuynia cordata* Thunb as Insecticide/Repellent against *Solenopsis invicta* and Repositioning Plant Fungicides against *Colletotrichum fragariae*

Aigerim Kurmanbayeva [1,2,†], Meirambek Ospanov [1,†], Prabin Tamang [3], Farhan Mahmood Shah [1], Abbas Ali [1], Zeyad M. A. Ibrahim [4], Charles L. Cantrell [3], Satmbekova Dinara [2,5], Ubaidilla Datkhayev [2,*], Ikhlas A. Khan [1] and Mohamed A. Ibrahim [1,*]

1 National Center for Natural Products Research, School of Pharmacy, University of Mississippi, University, MS 38677, USA; aigerimkurmanbaeva14@gmail.com (A.K.); mospanov@olemiss.edu (M.O.); fshah@olemiss.edu (F.M.S.); aali@olemiss.edu (A.A.); ikhan@olemiss.edu (I.A.K.)
2 Department of Organization, Management and Economics of Pharmacy and Clinical Pharmacy, School of Pharmacy, S.D. Asfendiyarov Kazakh National Medical University, Almaty 050012, Kazakhstan; dskanatovna@gmail.com
3 USDA-ARS, Natural Products Utilization Research Unit, University, MS 38677, USA; prabin.tamang@usda.gov (P.T.); charles.cantrell@usda.gov (C.L.C.)
4 General Studies, University of Mississippi, University, MS 38677, USA; zmibrahi@go.olemiss.edu
5 The National Academy of Science, Republic of Kazakhstan, Almaty 050010, Kazakhstan
* Correspondence: u.datkhayev@kaznmu.kz (U.D.); mmibrahi@olemiss.edu (M.A.I.)
† These authors contributed equally to this work.

Supplementary Information

Page Number	Figure
Figure S1	^{1}H NMR spectrum of **1**
Figure S2	^{13}C NMR spectrum of **1**
Figure S3	Dept-135 NMR spectrum of **1**
Figure S4	^{1}H NMR spectrum of **2a**
Figure S5	^{13}C NMR spectrum of **2a**
Figure S6	Dept-135 NMR spectrum of **2a**
Figure S7	HRMS of **2a**
Figure S8	^{1}H NMR spectrum of **2b**
Figure S9	^{13}C NMR spectrum of **2b**
Figure S10	Dept-135 NMR spectrum of **2b**
Figure S11	HRMS of **2b**
Figure S12	^{1}H NMR spectrum of **2c**
Figure S13	^{13}C NMR spectrum of **2c**
Figure S14	Dept-135 NMR spectrum of **2c**
Figure S15	HRMS of **2c**
Figure S16	^{1}H NMR spectrum of **2d**

Page Number	Figure
Figure S17	^{13}C NMR spectrum of **2d**
Figure S18	Dept-135 NMR spectrum of **2d**
Figure S19	HRMS of **2d**
Figure S20	^{1}H NMR spectrum of **2e**
Figure S21	^{13}C NMR spectrum of **2e**
Figure S22	Dept-135 NMR spectrum of **2e**
Figure S23	HRMS of **2e**
Figure S24	^{1}H NMR spectrum of **2f**
Figure S25	^{13}C NMR spectrum of **2f**
Figure S26	Dept-135 NMR spectrum of **2f**
Figure S27	HRMS of **2f**

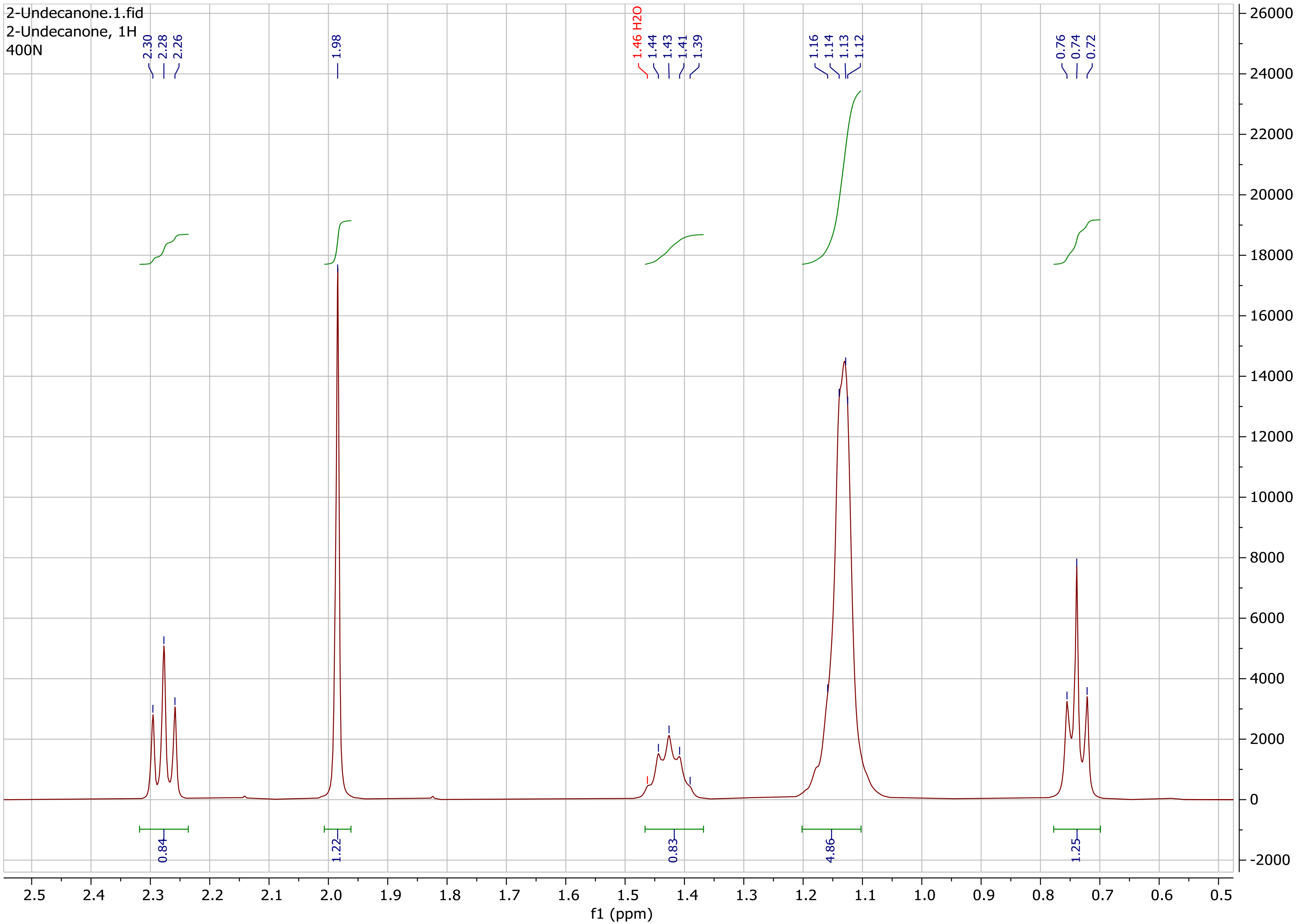

Figure S1: ^{1}H NMR spectrum of **1** [400 MHz, $CDCl_3$]

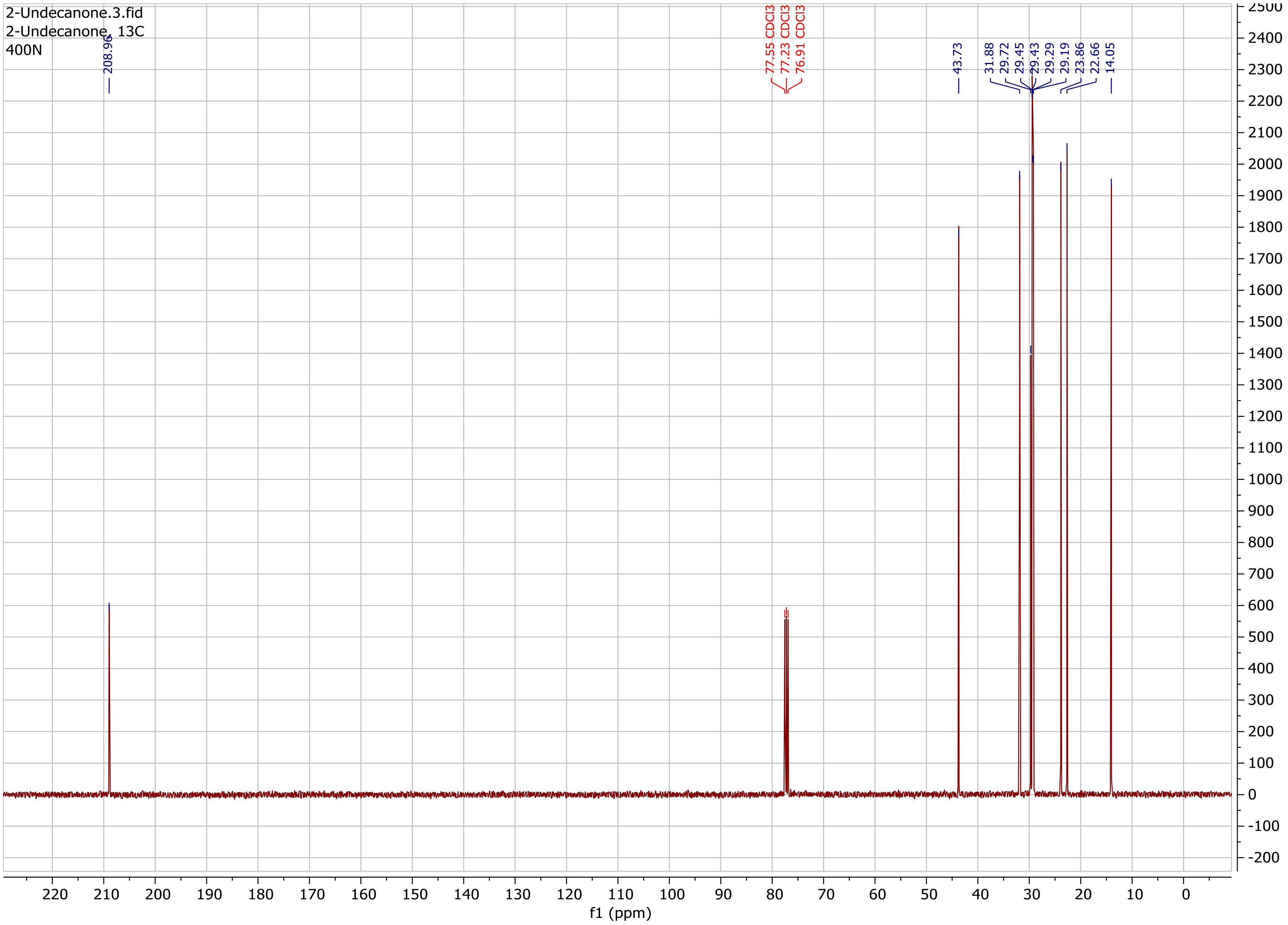

Figure S2: ^{13}C NMR spectrum of **1** [100 MHz, $CDCl_3$]

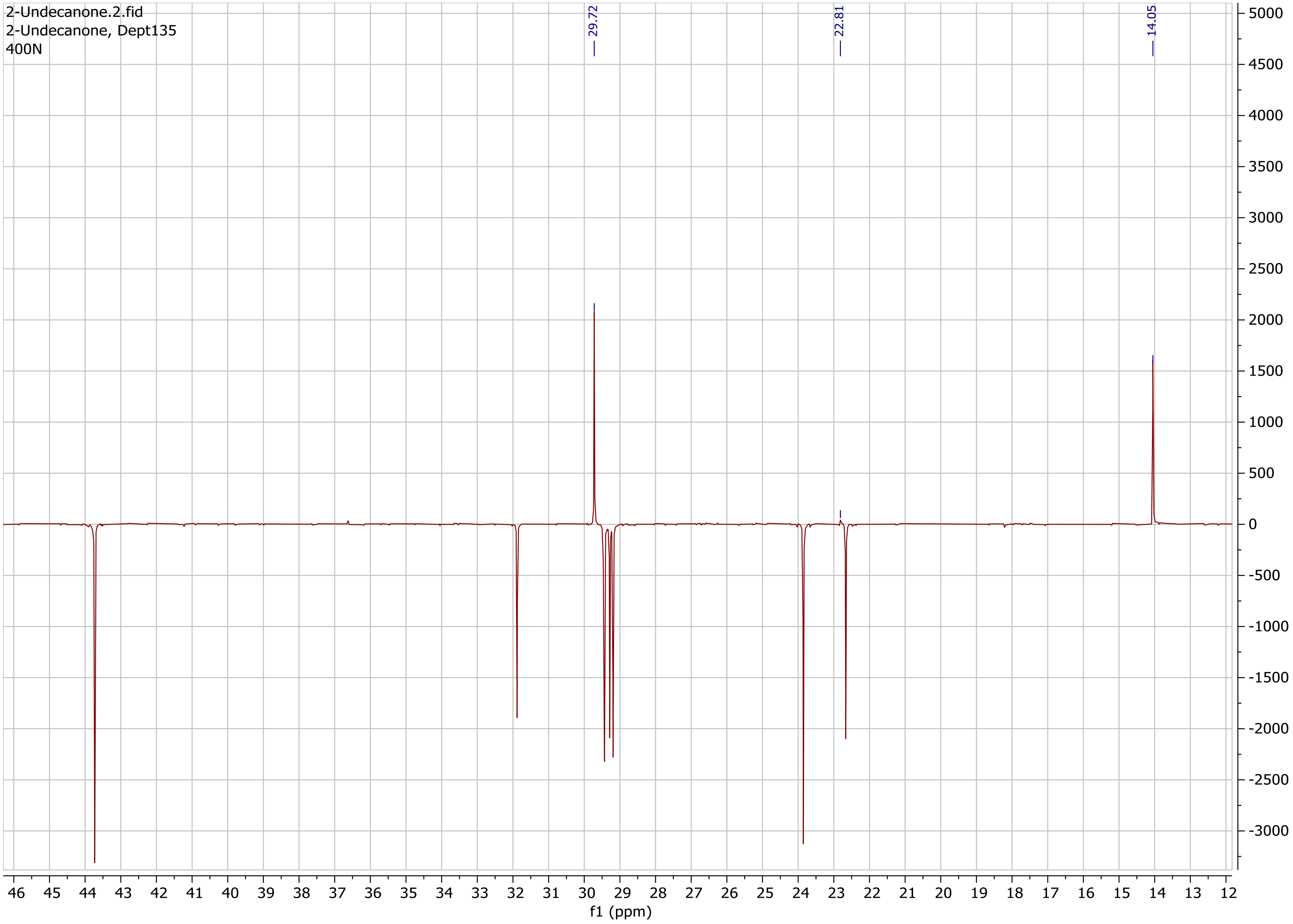

Figure S3: Dept-135 NMR spectrum of **1** [400 MHz, $CDCl_3$]

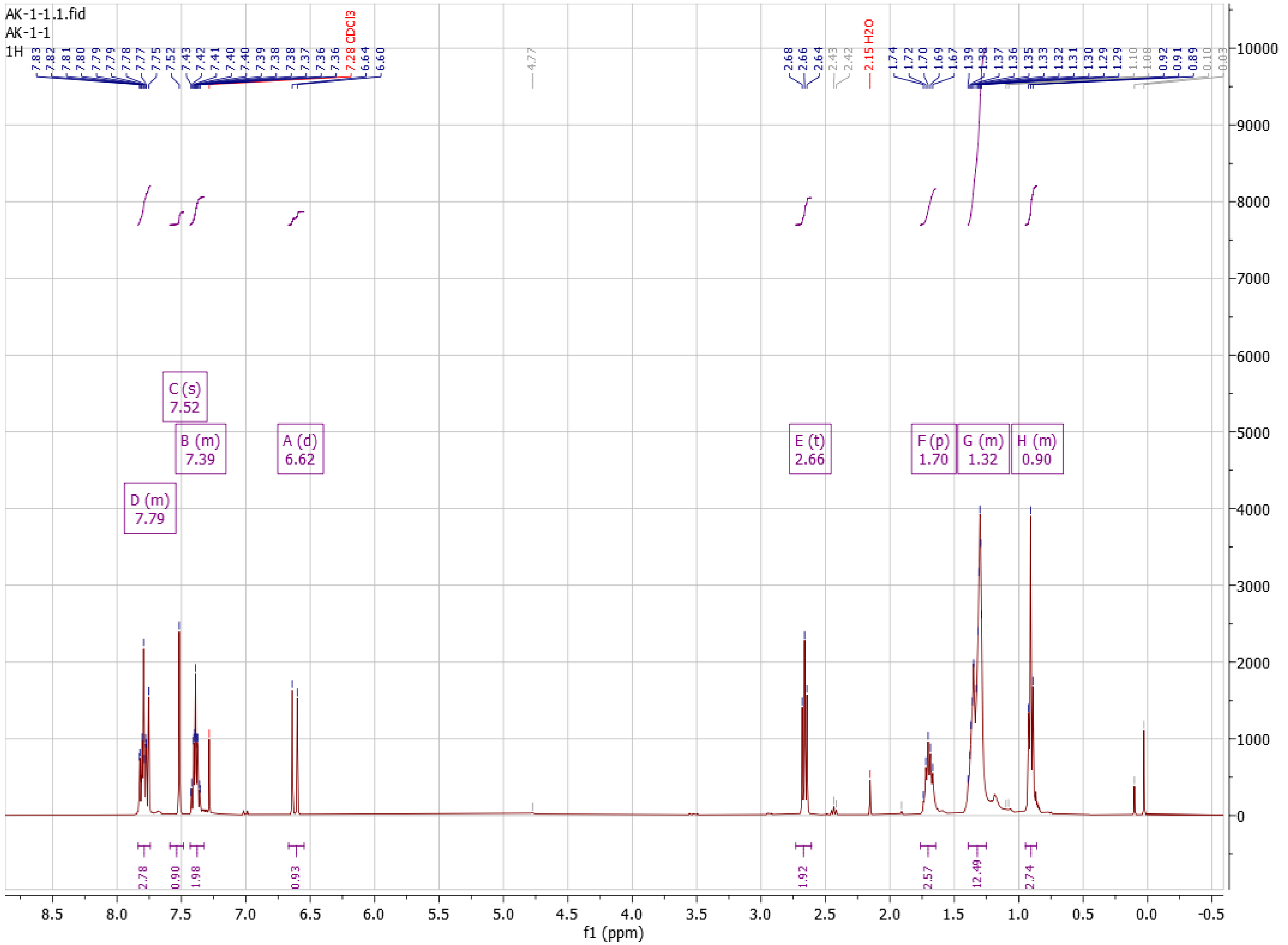

Figure S4: ^{1}H NMR spectrum of **2a** [400 MHz, $CDCl_3$]

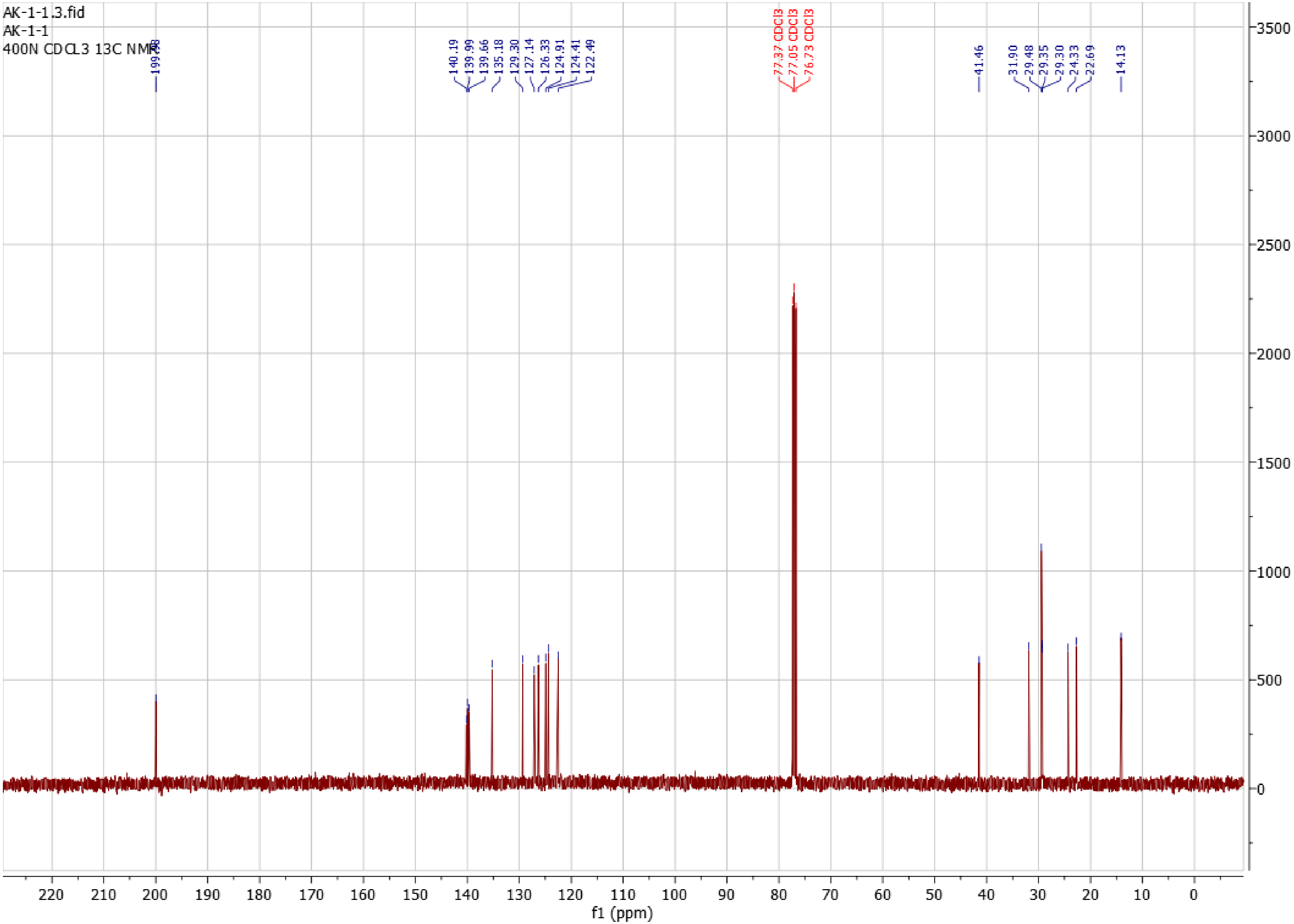

Figure S5: ^{13}C NMR spectrum of **2a** [100 MHz, $CDCl_3$]

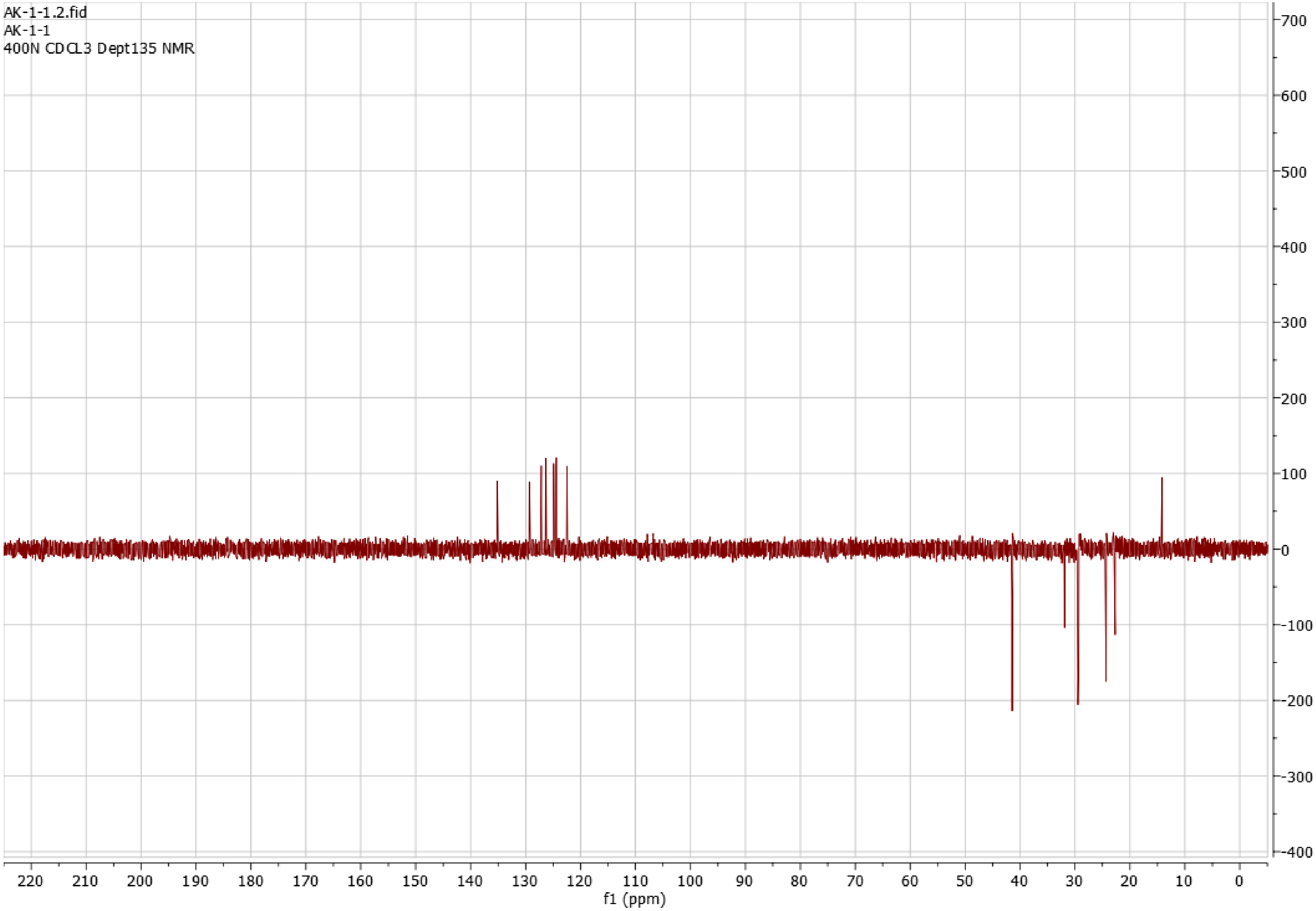

Figure S6: Dept-135 NMR spectrum of **2a** [400 MHz, $CDCl_3$]

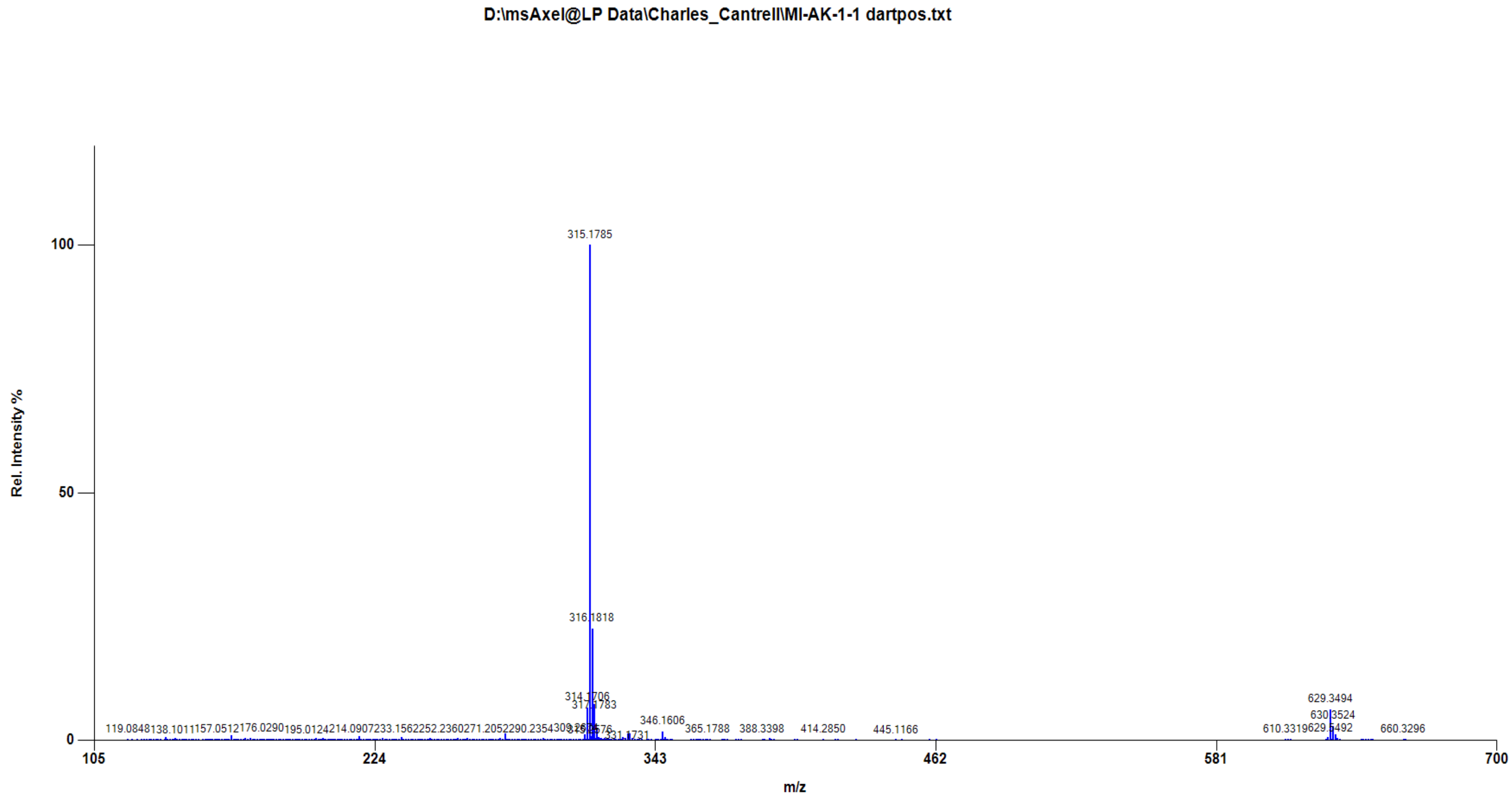

Figure S7: HRMS of **2a**

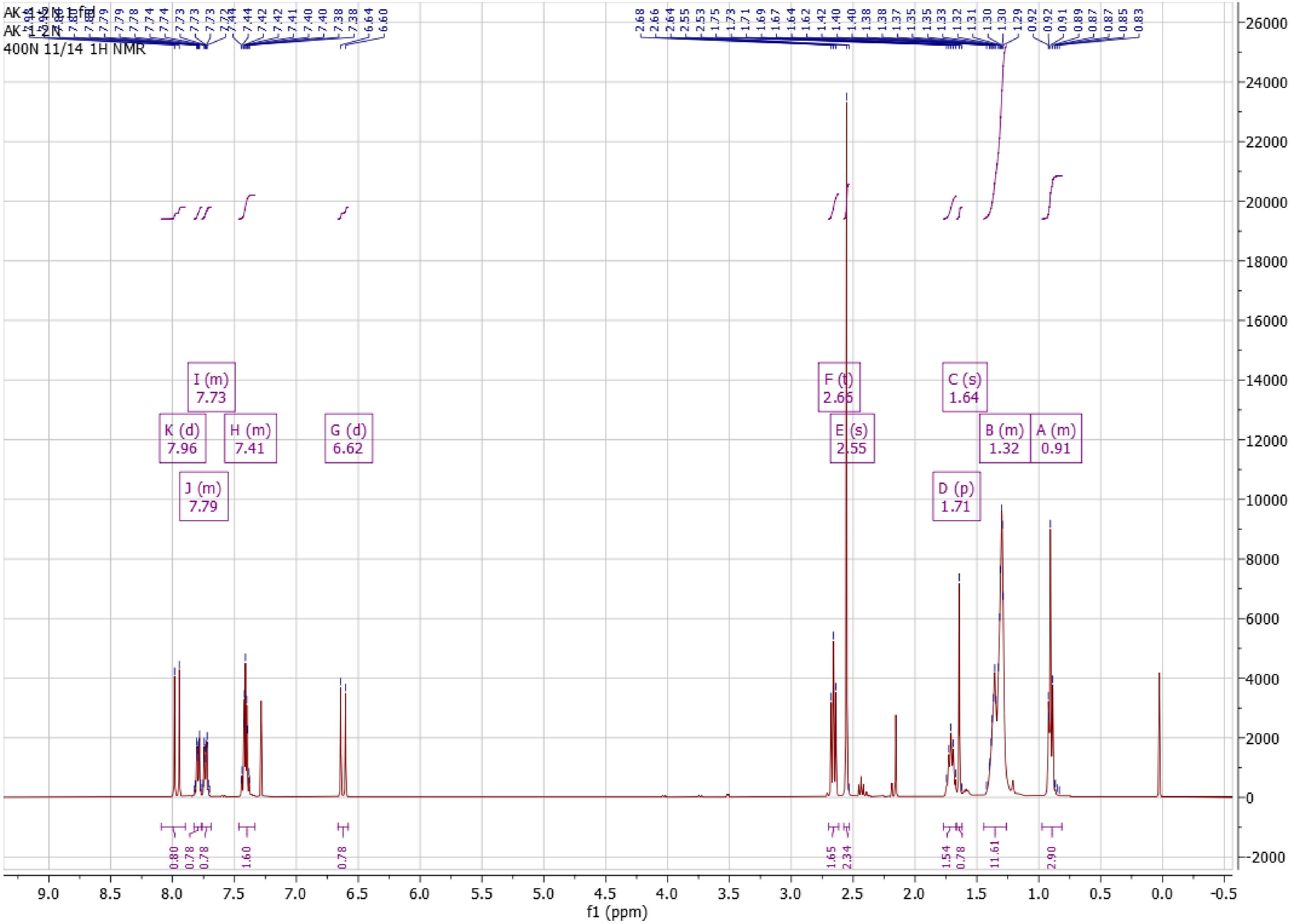

Figure S8: ^{1}H NMR spectrum of **2b** [400 MHz, $CDCl_3$]

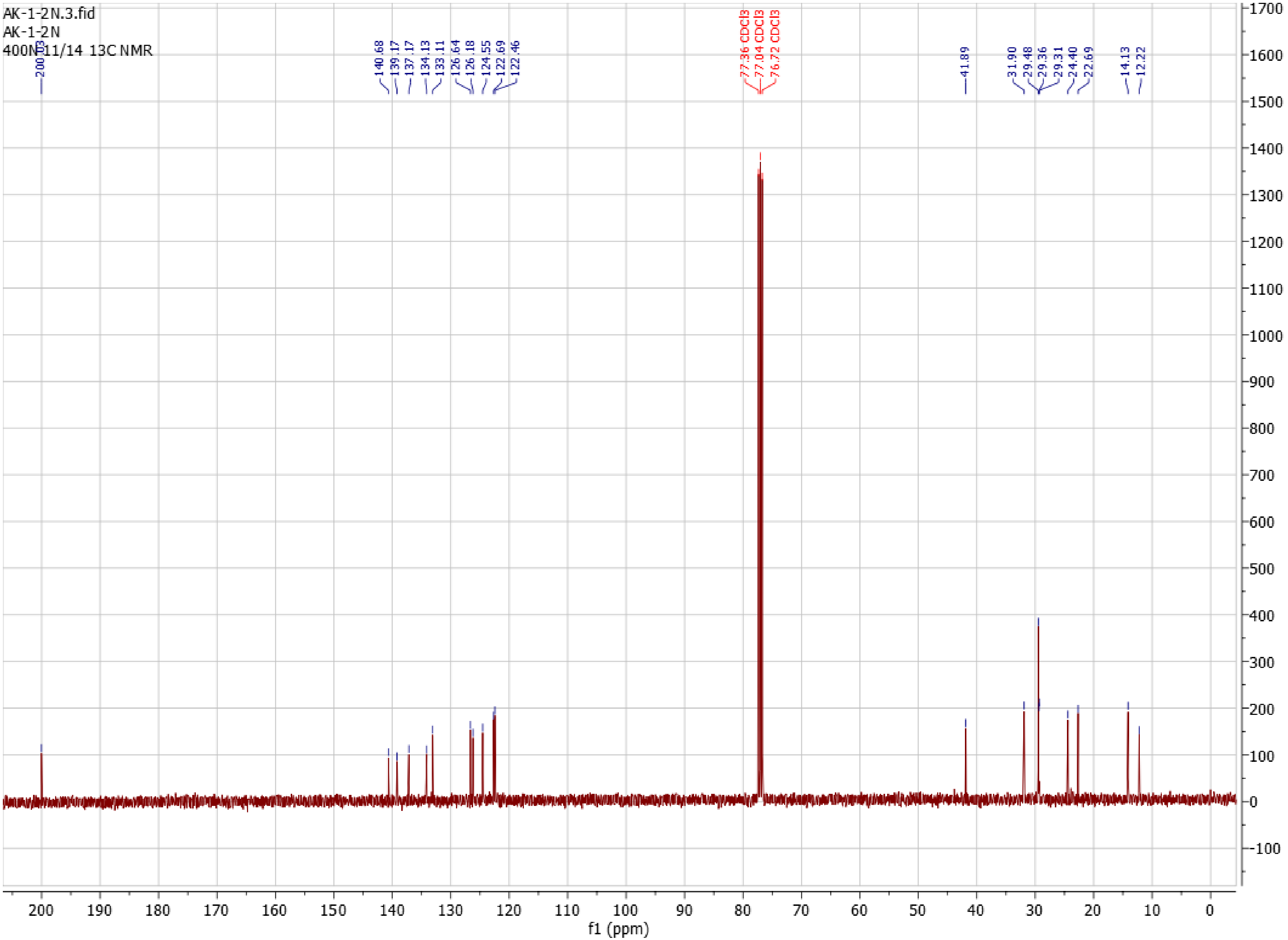

Figure S9: ^{13}C NMR spectrum of **2b** [100 MHz, $CDCl_3$]

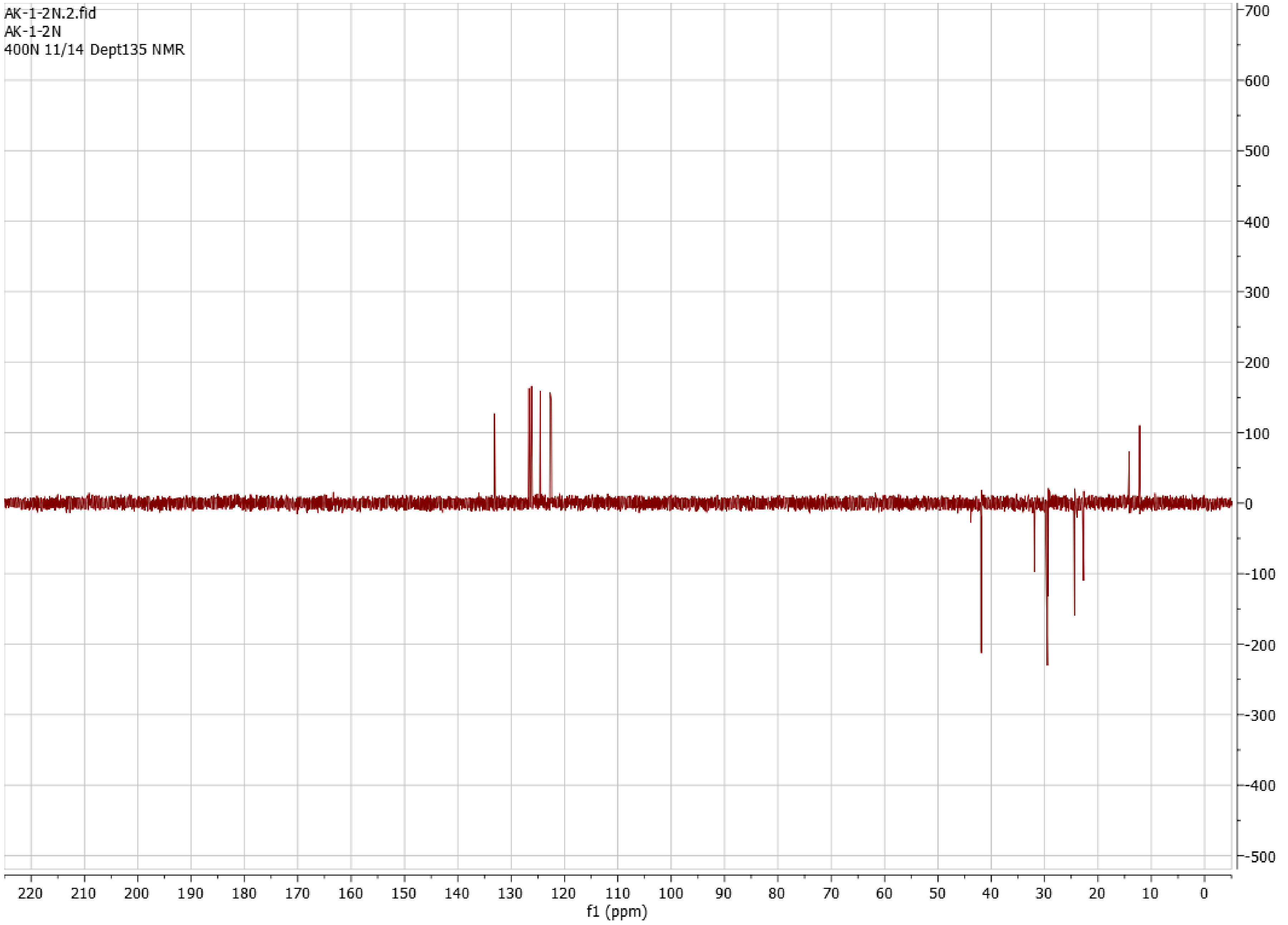

Figure S10: Dept-135 NMR spectrum of **2b** [400 MHz, $CDCl_3$]

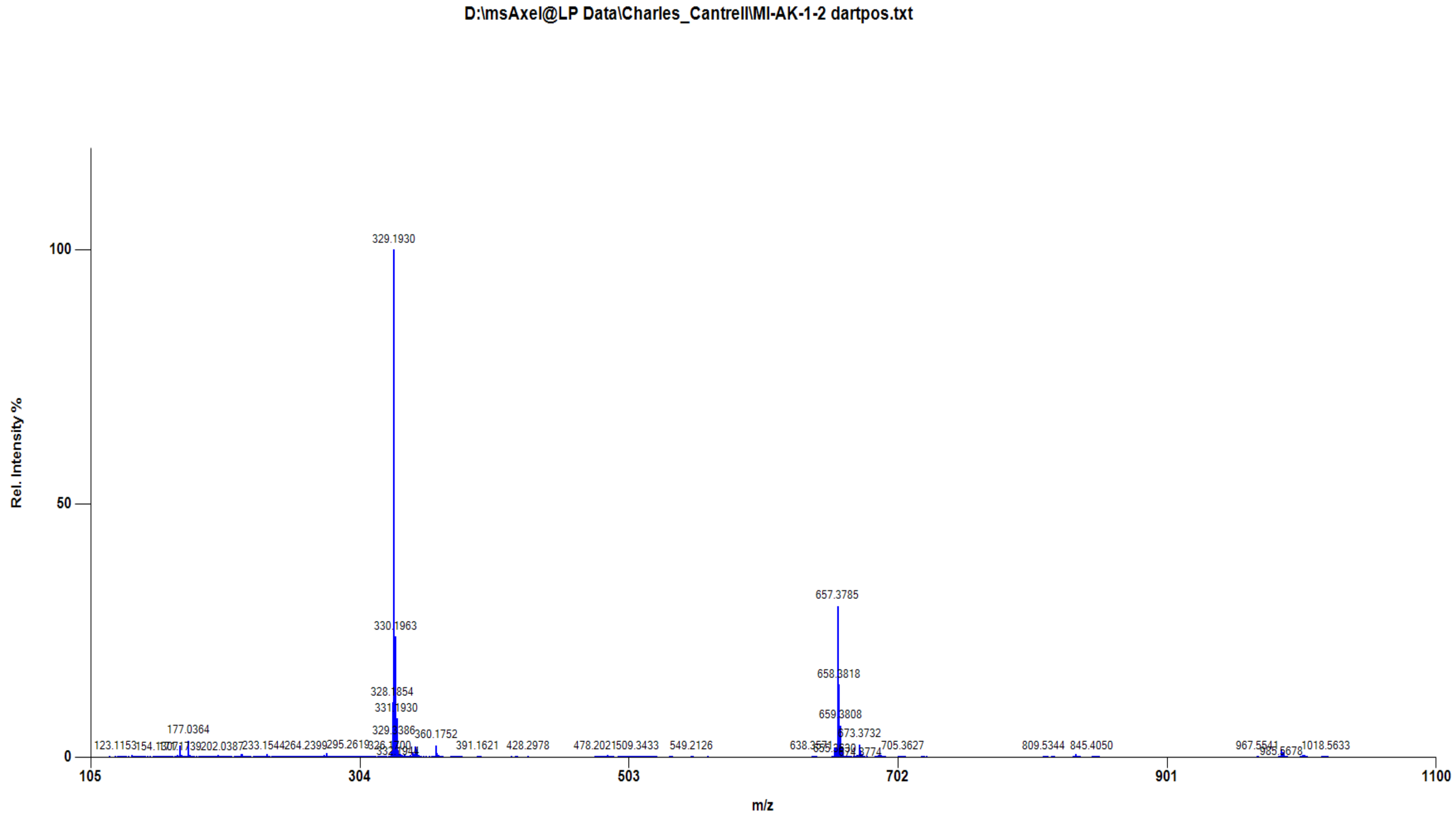

Figure S11: HRMS of **2b**

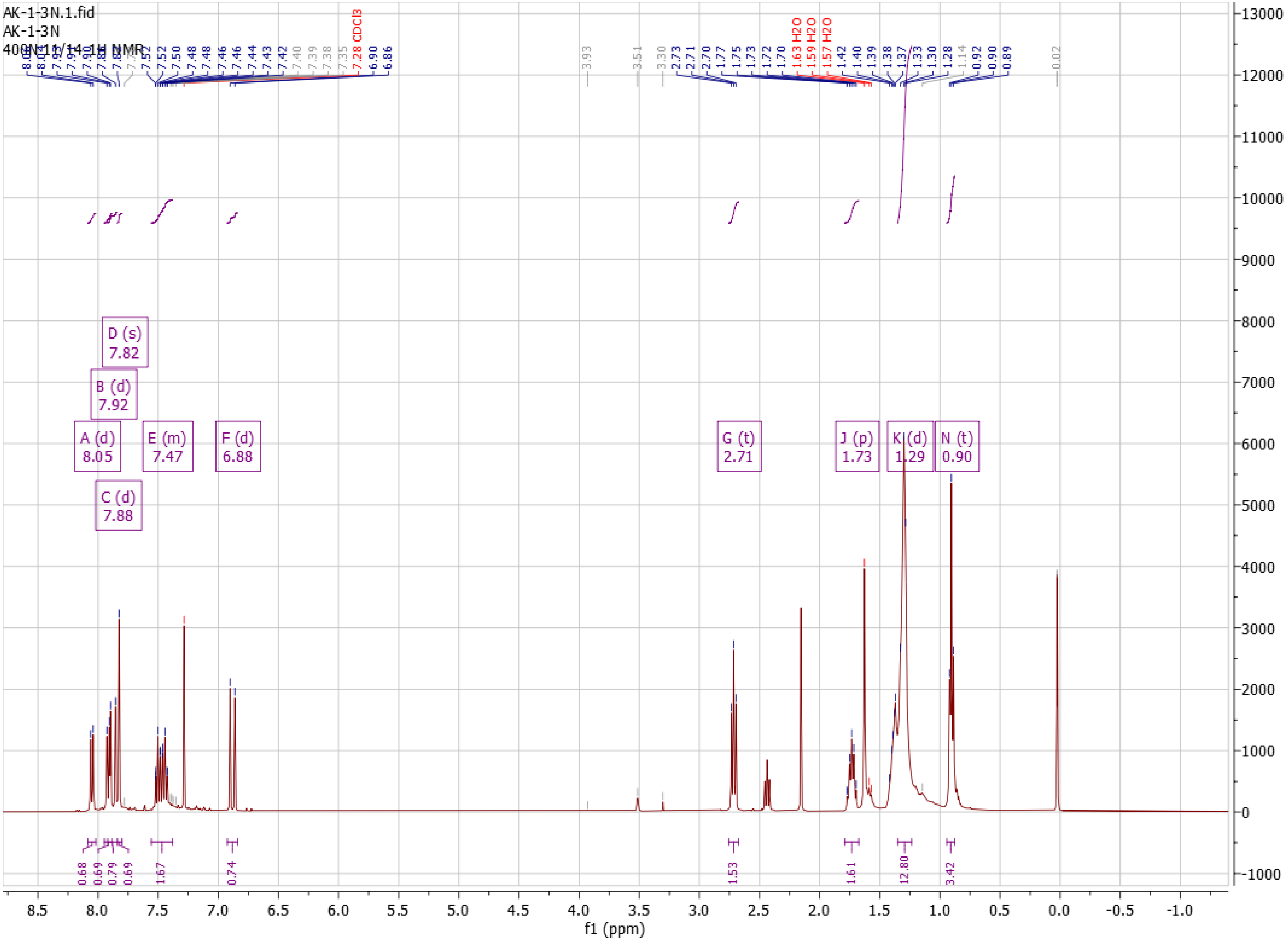

Figure S12: ^{1}H NMR spectrum of **2c** [400 MHz, $CDCl_3$]

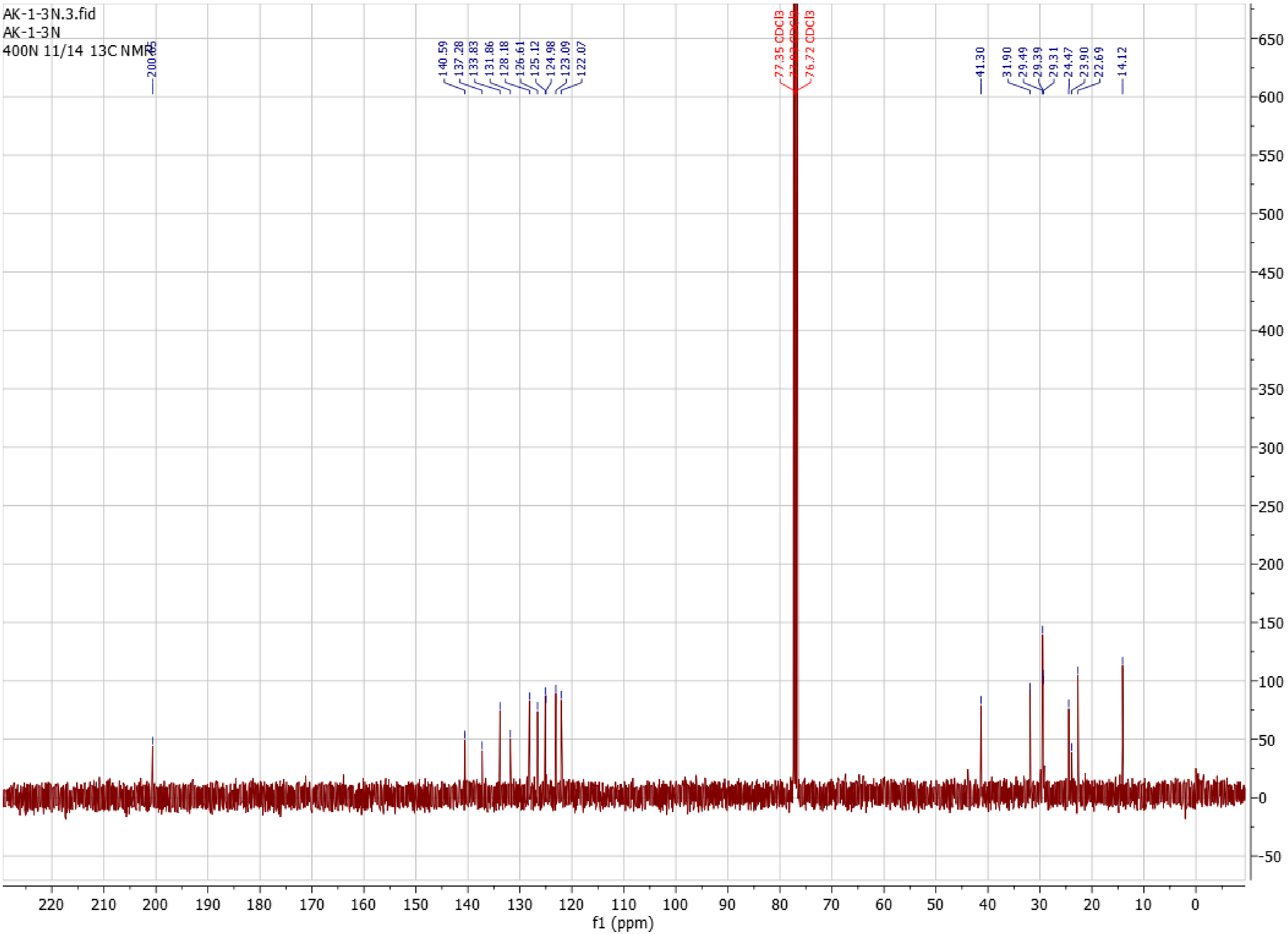

Figure S13: ^{13}C NMR spectrum of **2c** [100 MHz, $CDCl_3$]

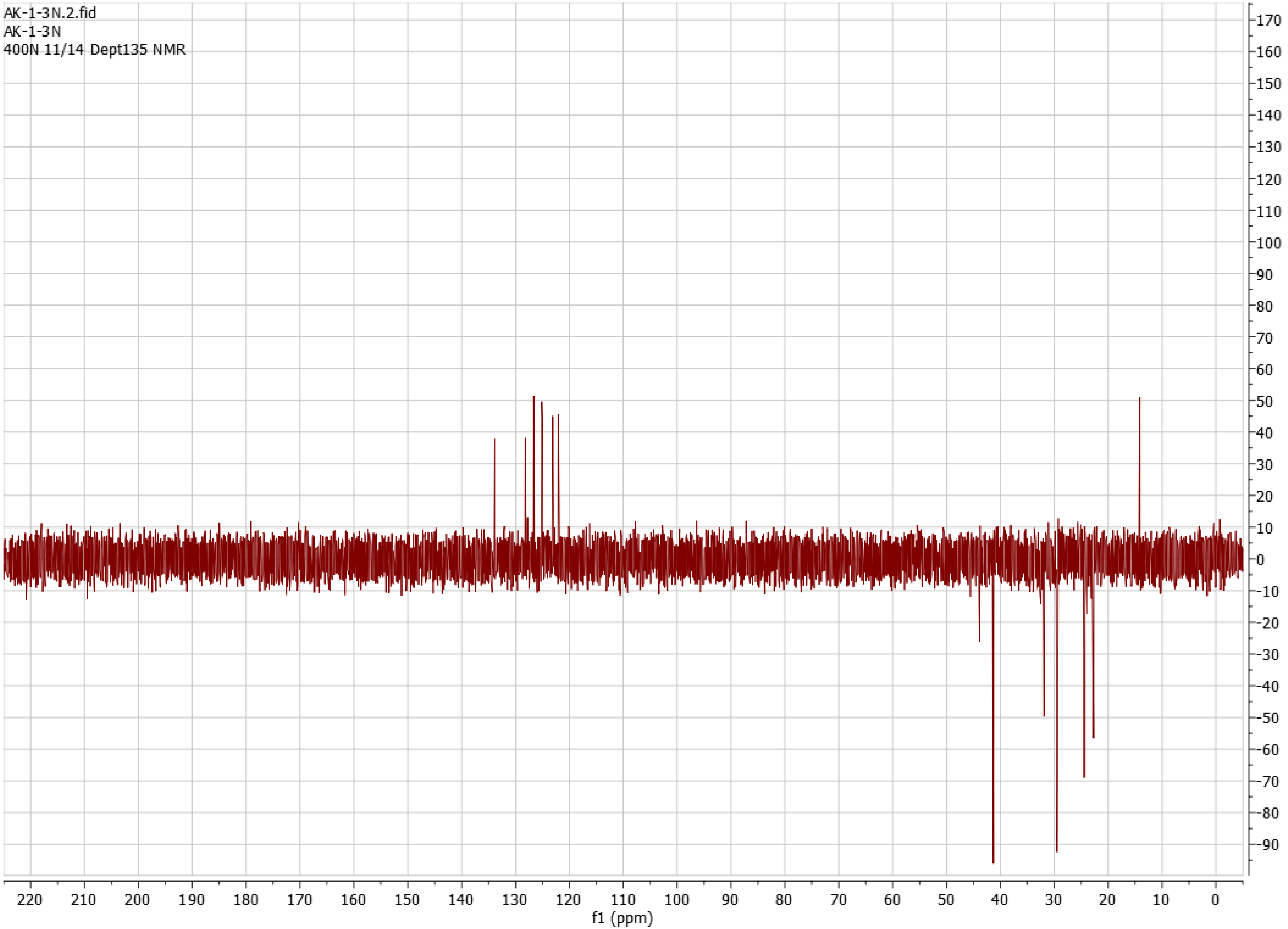

Figure S14: Dept-135 NMR spectrum of **2c** [400 MHz, $CDCl_3$]

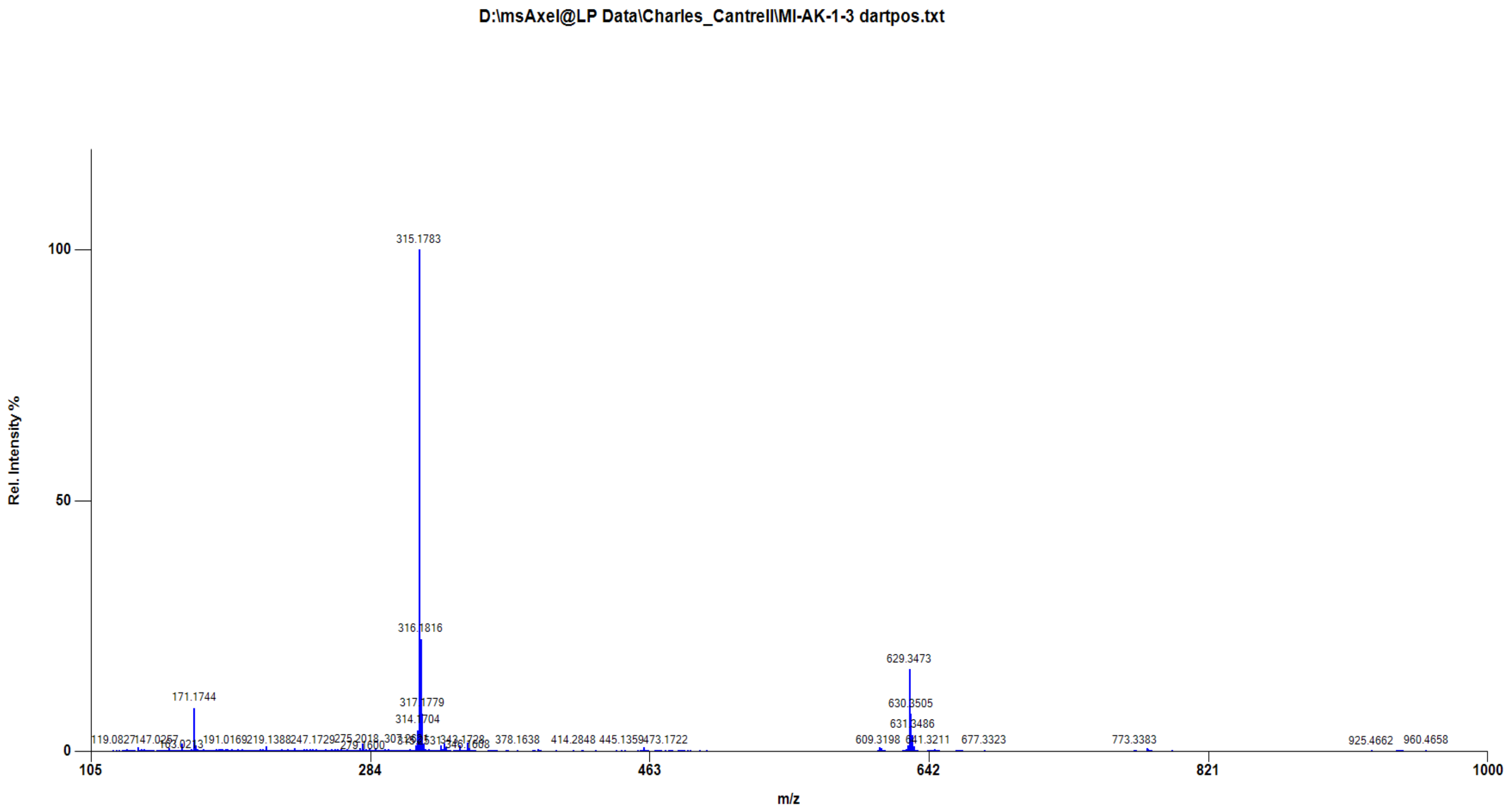

Figure S15: HRMS of **2c**

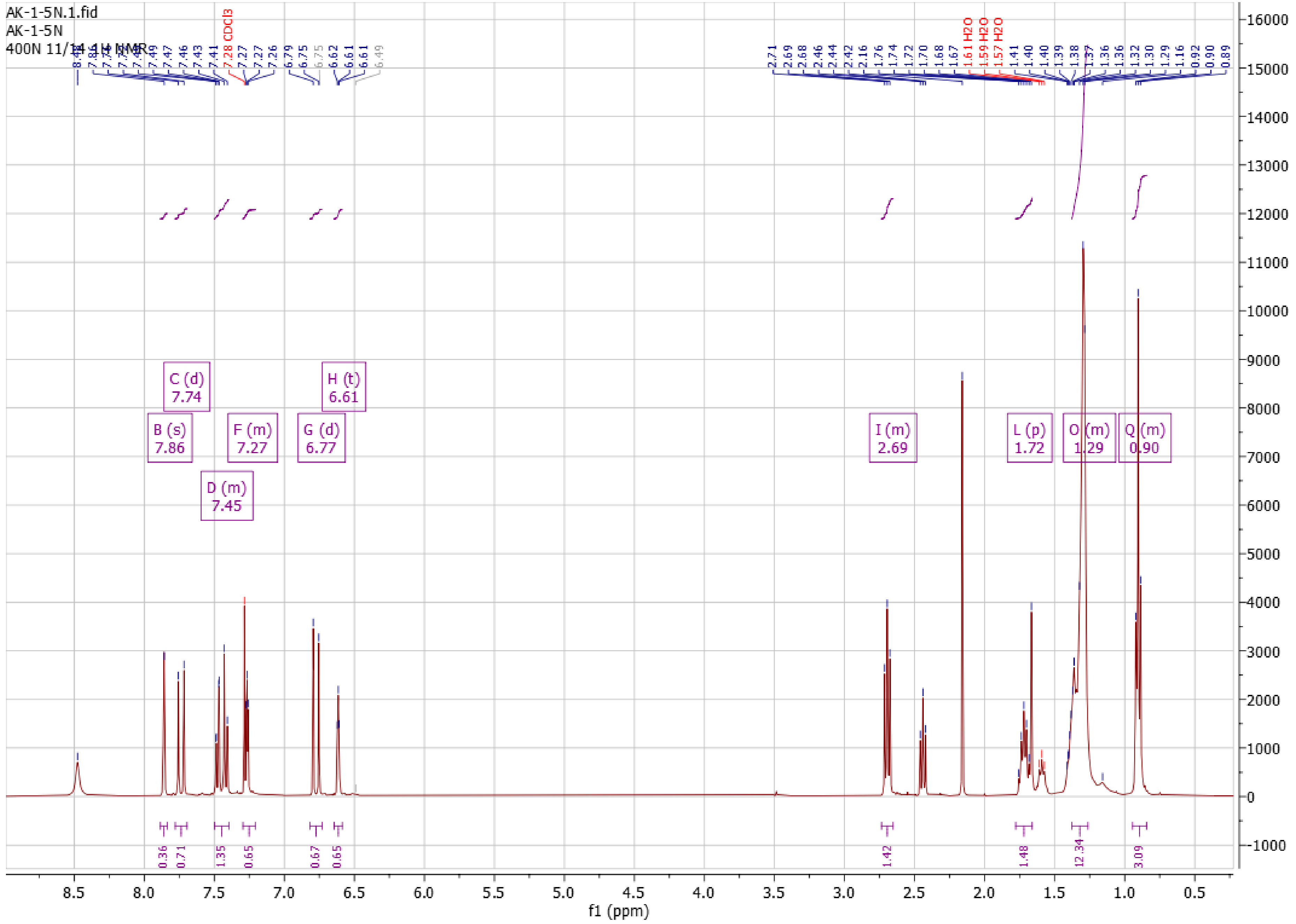

Figure S16: ^{1}H NMR spectrum of **2d** [400 MHz, $CDCl_3$]

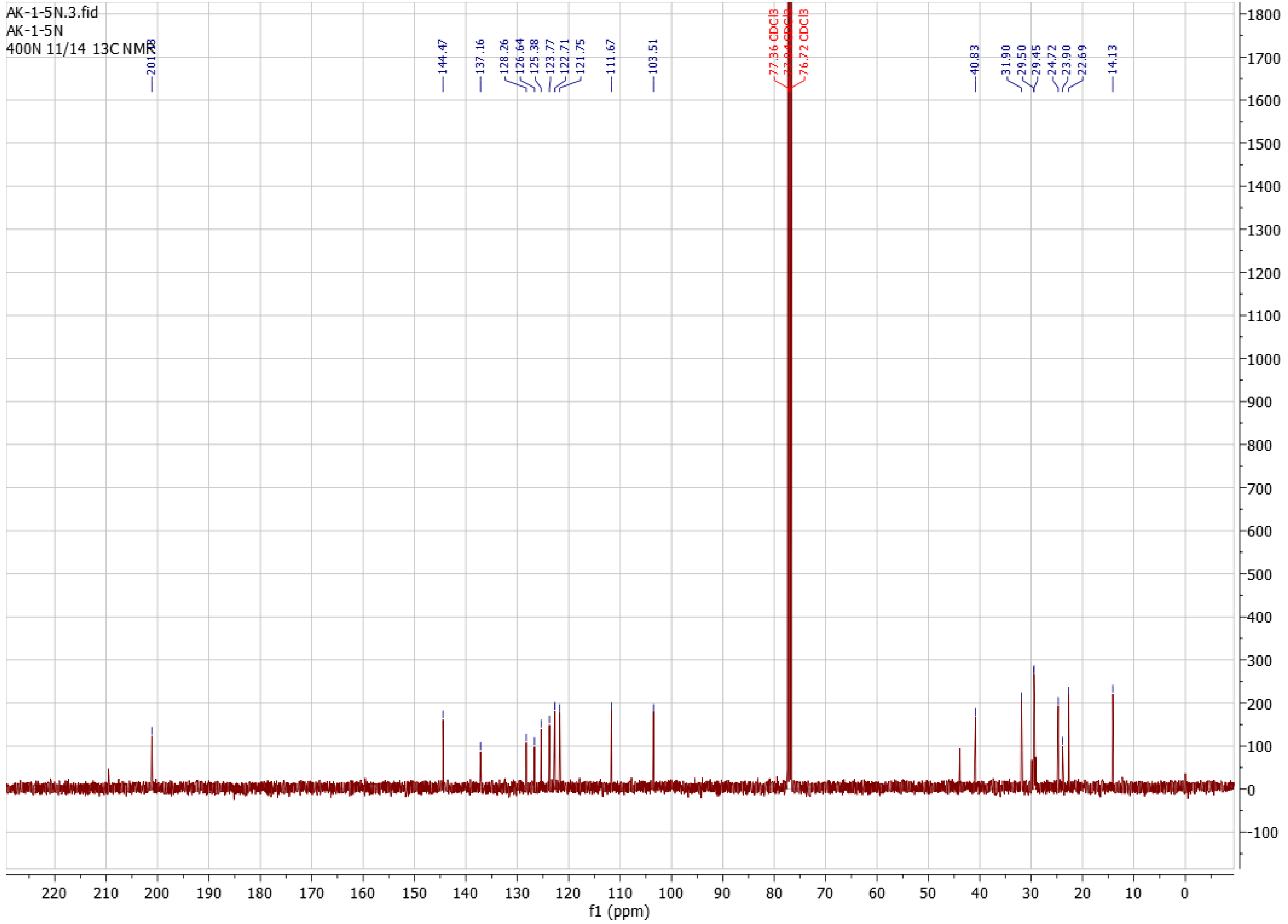

Figure S17: ^{13}C NMR spectrum of **2d** [100 MHz, $CDCl_3$]

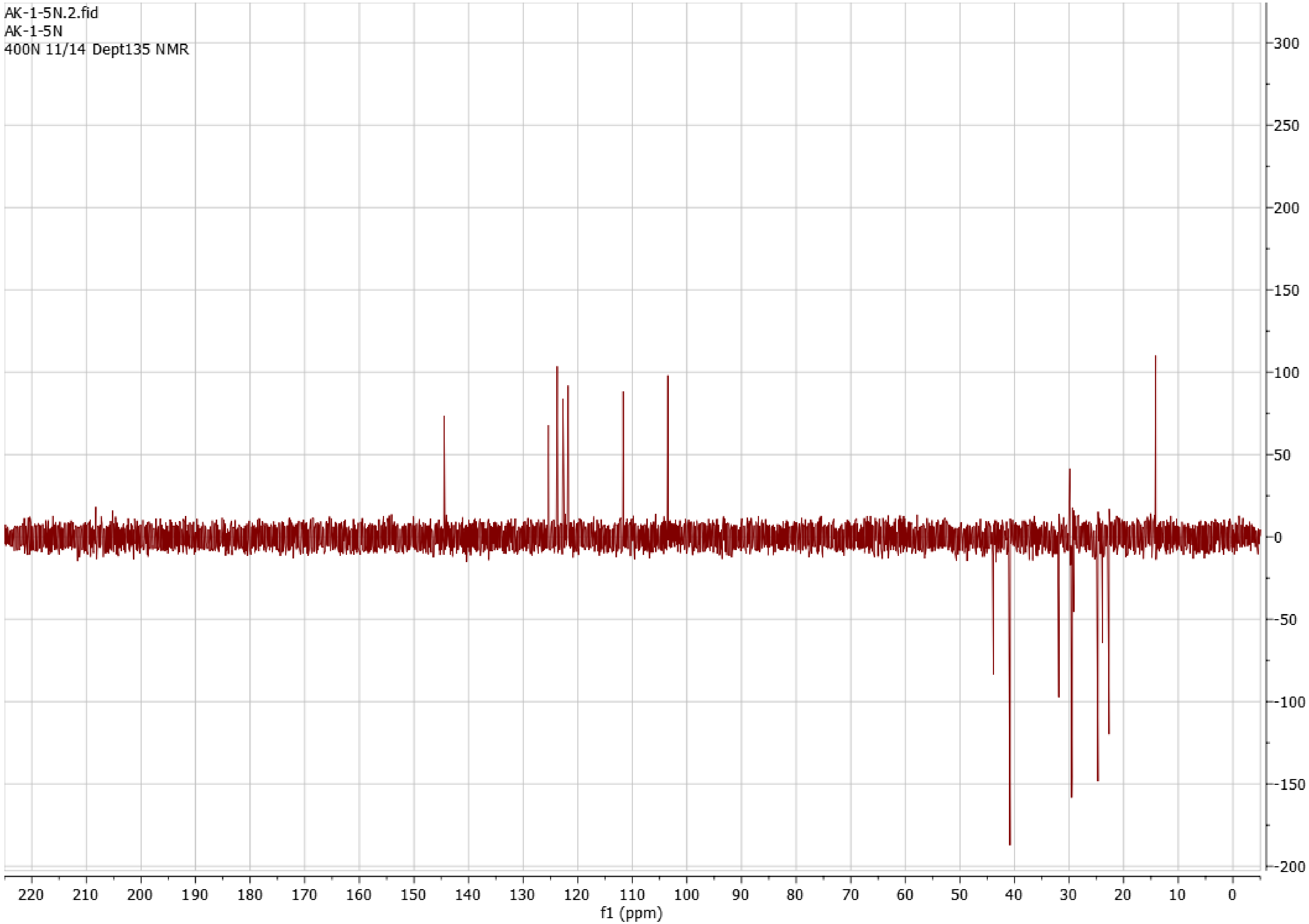

Figure S18: Dept-135 NMR spectrum of **2d** [400 MHz, $CDCl_3$]

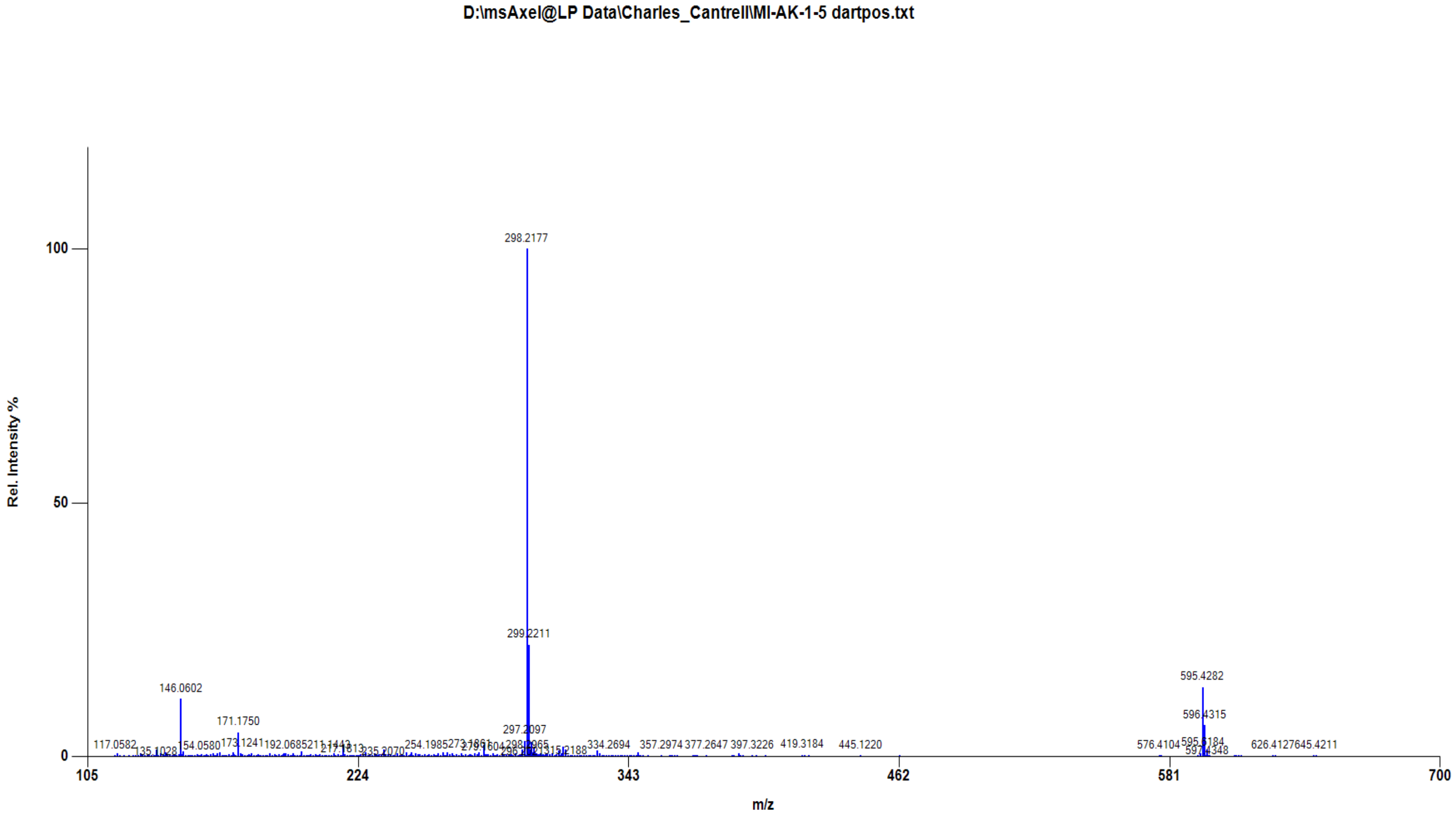

Figure S19: HRMS of **2d**

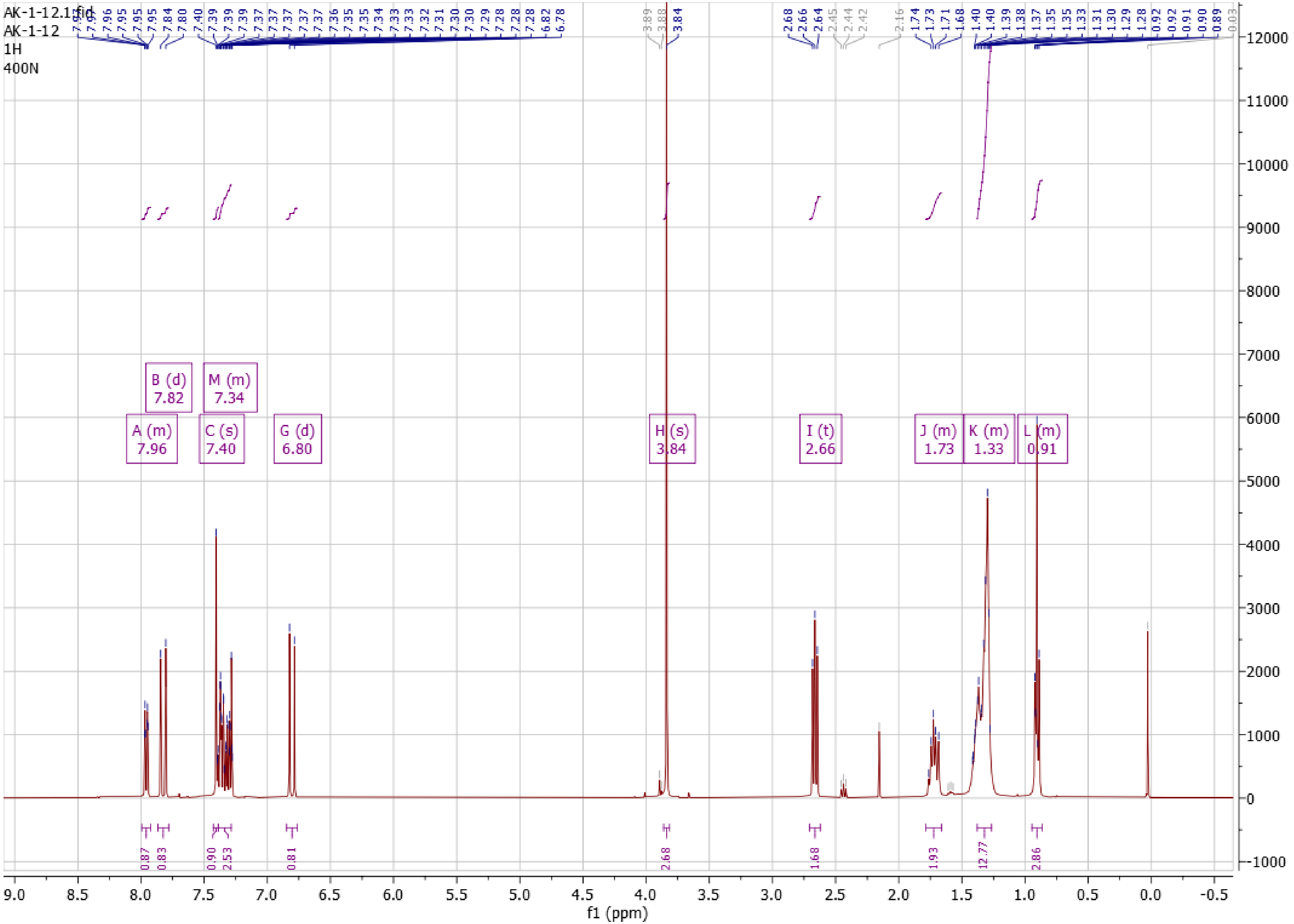

Figure S20: ^{1}H NMR spectrum of **2e** [400 MHz, $CDCl_3$]

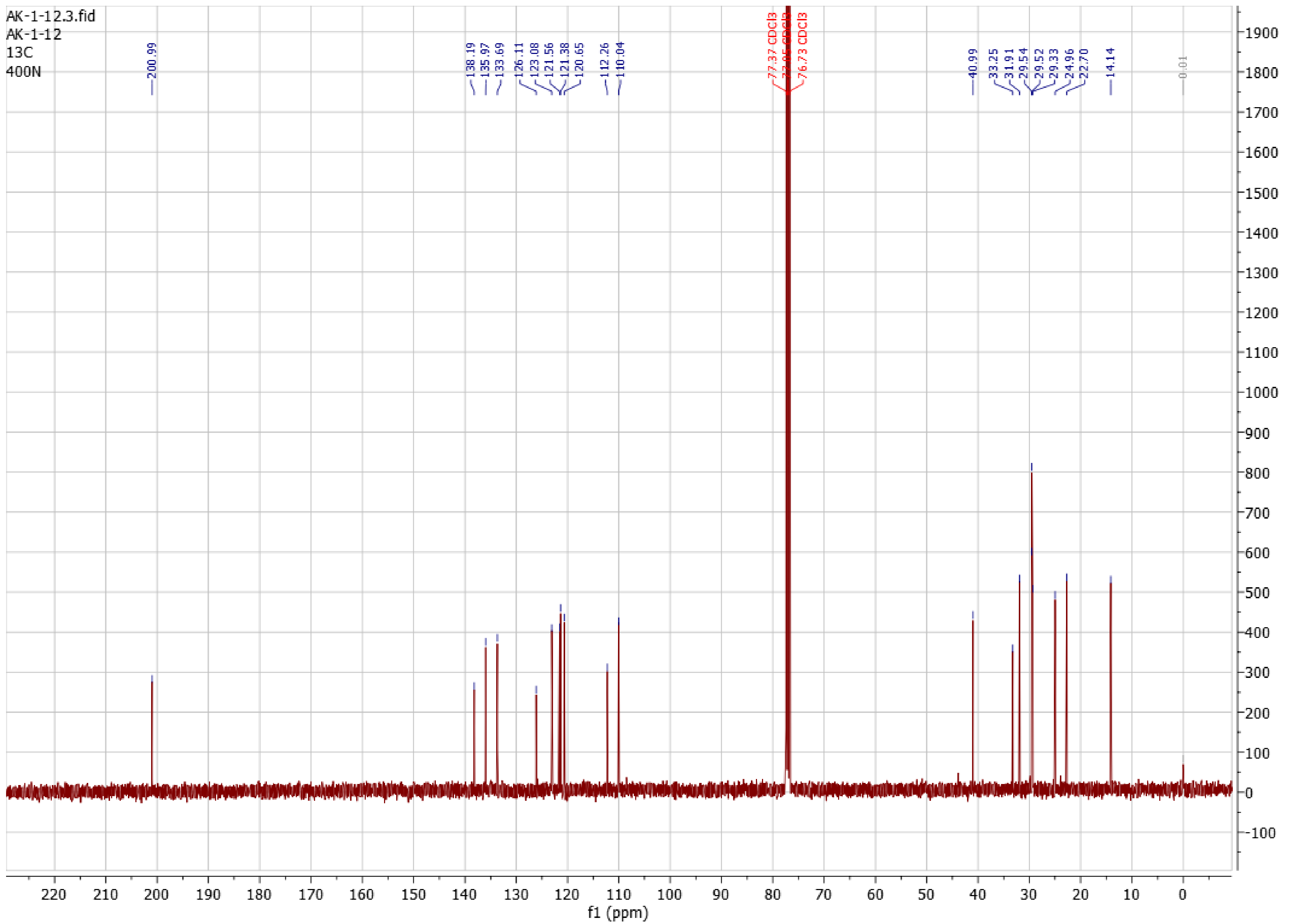

Figure S21: ^{13}C NMR spectrum of **2e** [100 MHz, $CDCl_3$]

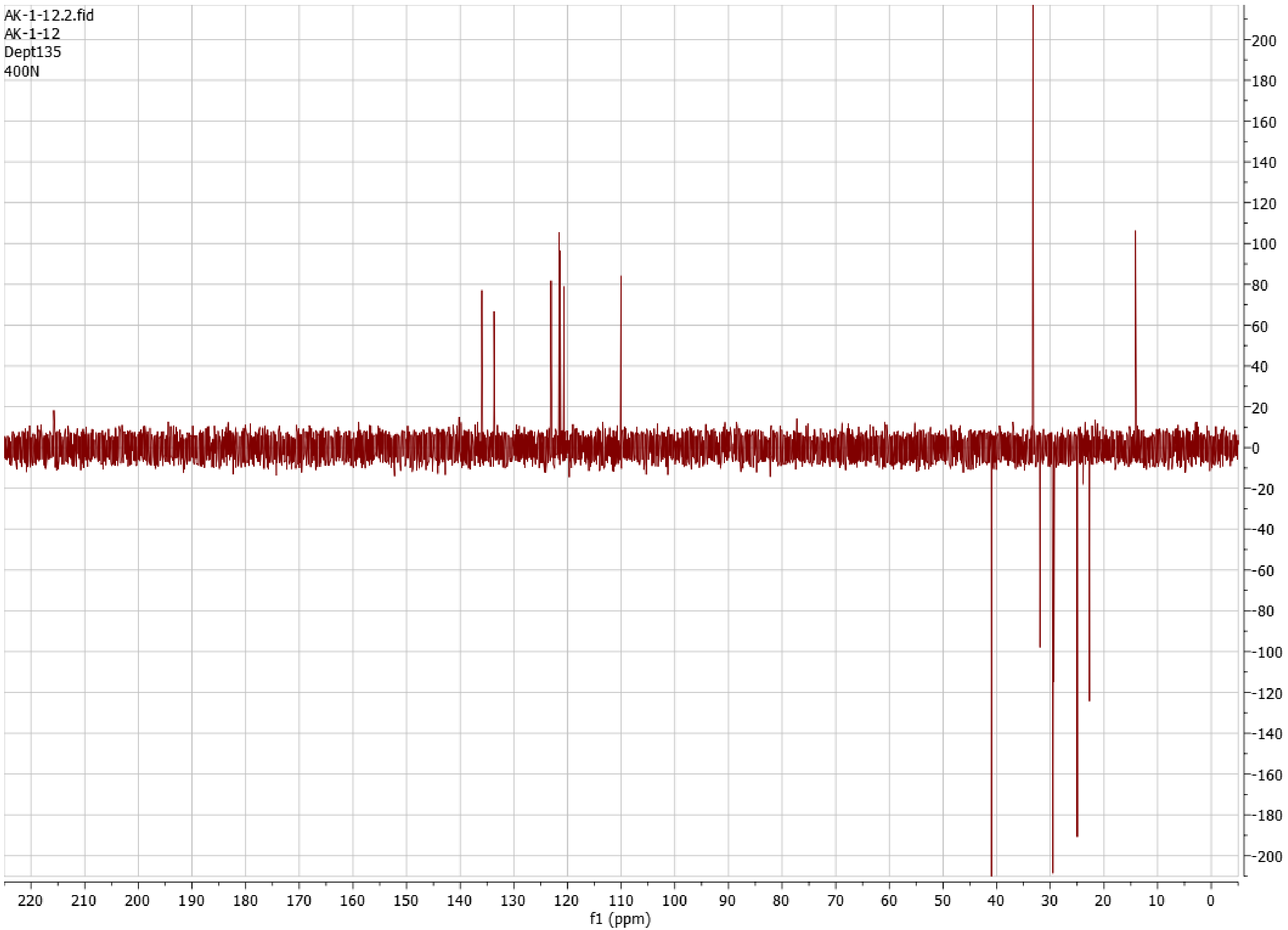

Figure S22: Dept-135 NMR spectrum of **2e** [400 MHz, $CDCl_3$]

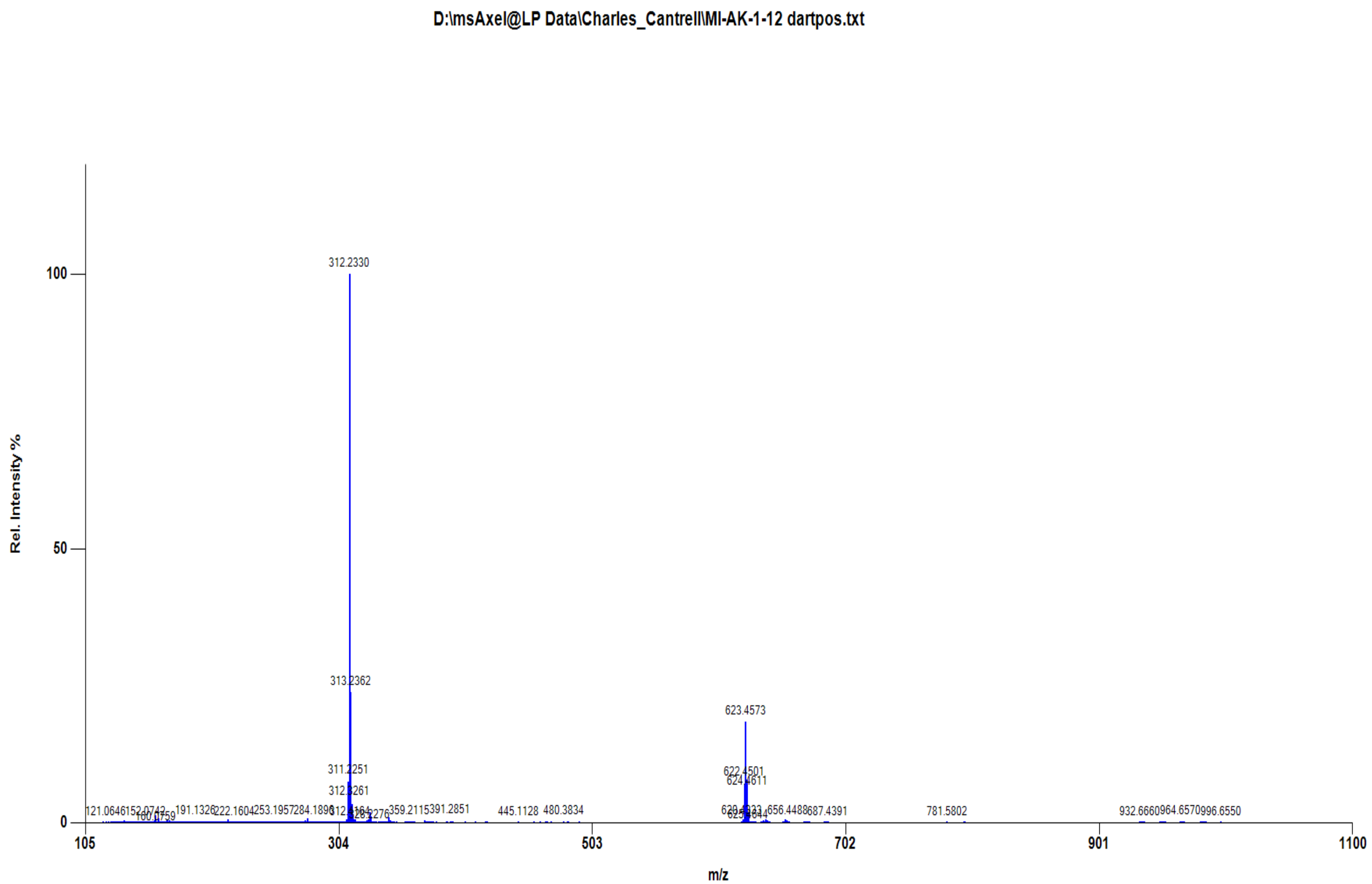

Figure S23: HRMS of **2e**

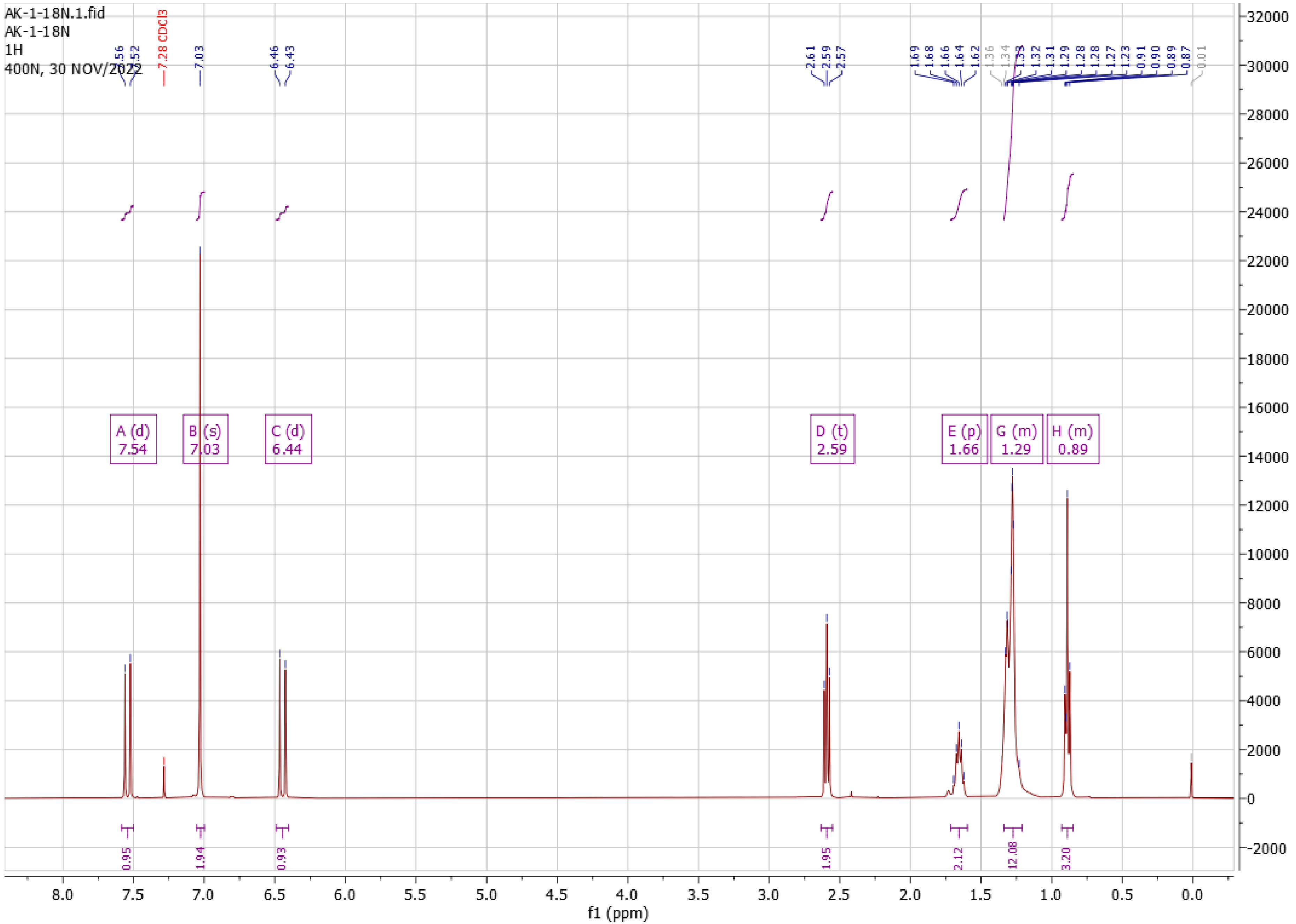

Figure S24: ^{1}H NMR spectrum of **2f** [400 MHz, $CDCl_3$]

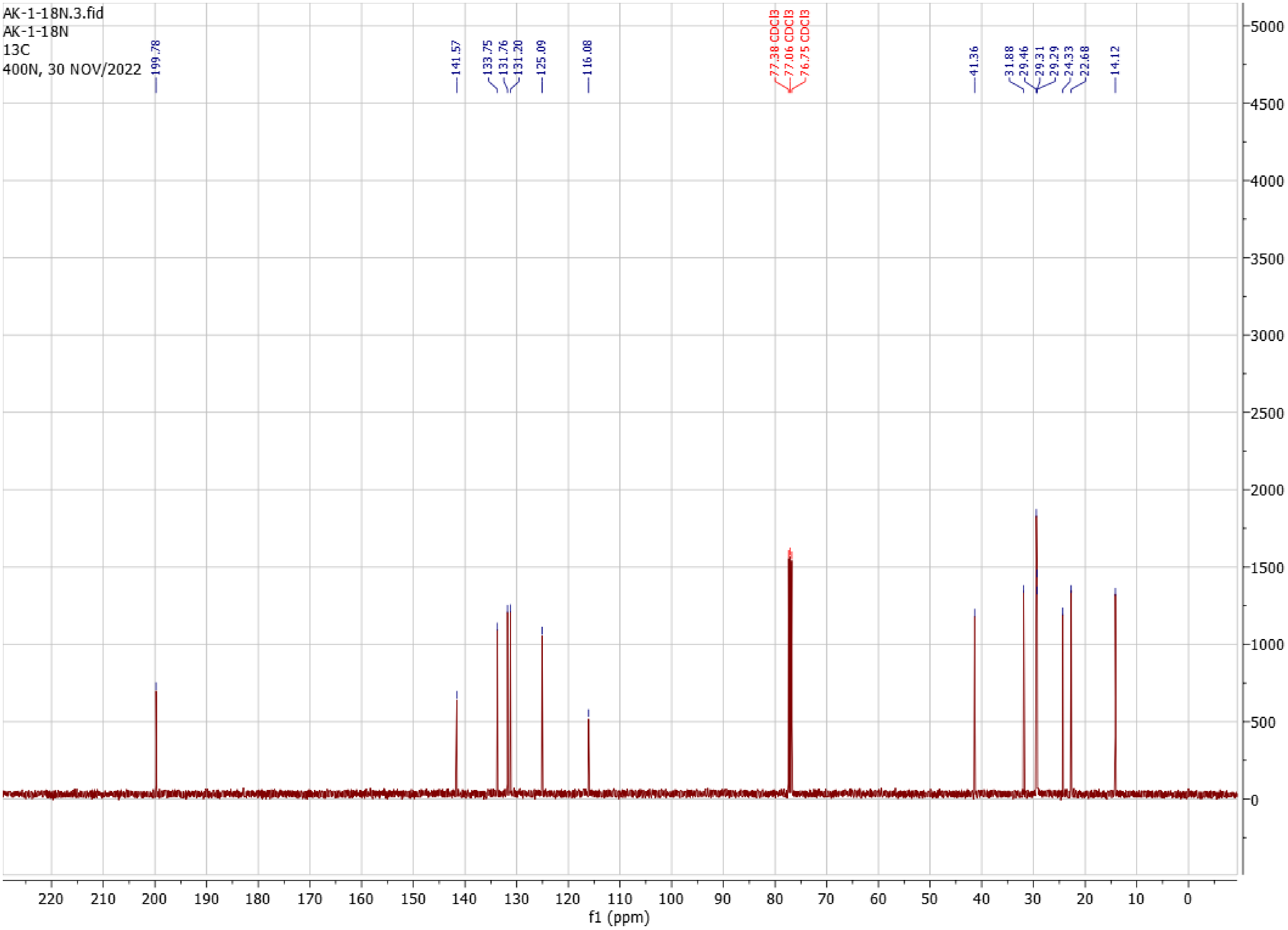

Figure S25: ^{13}C NMR spectrum of **2f** [100 MHz, $CDCl_3$]

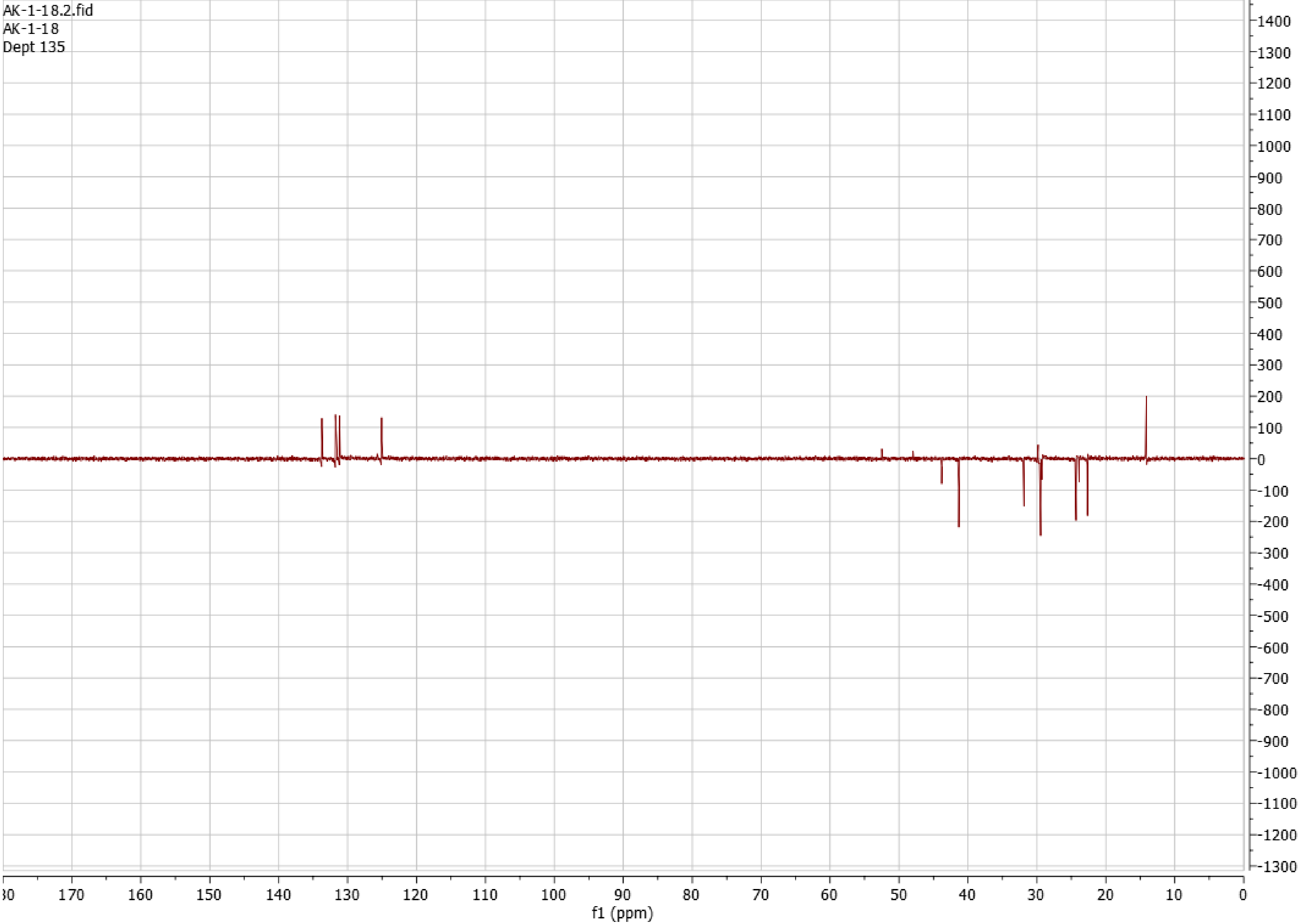

Figure S26: Dept-135 NMR spectrum of **2f** [400 MHz, $CDCl_3$]

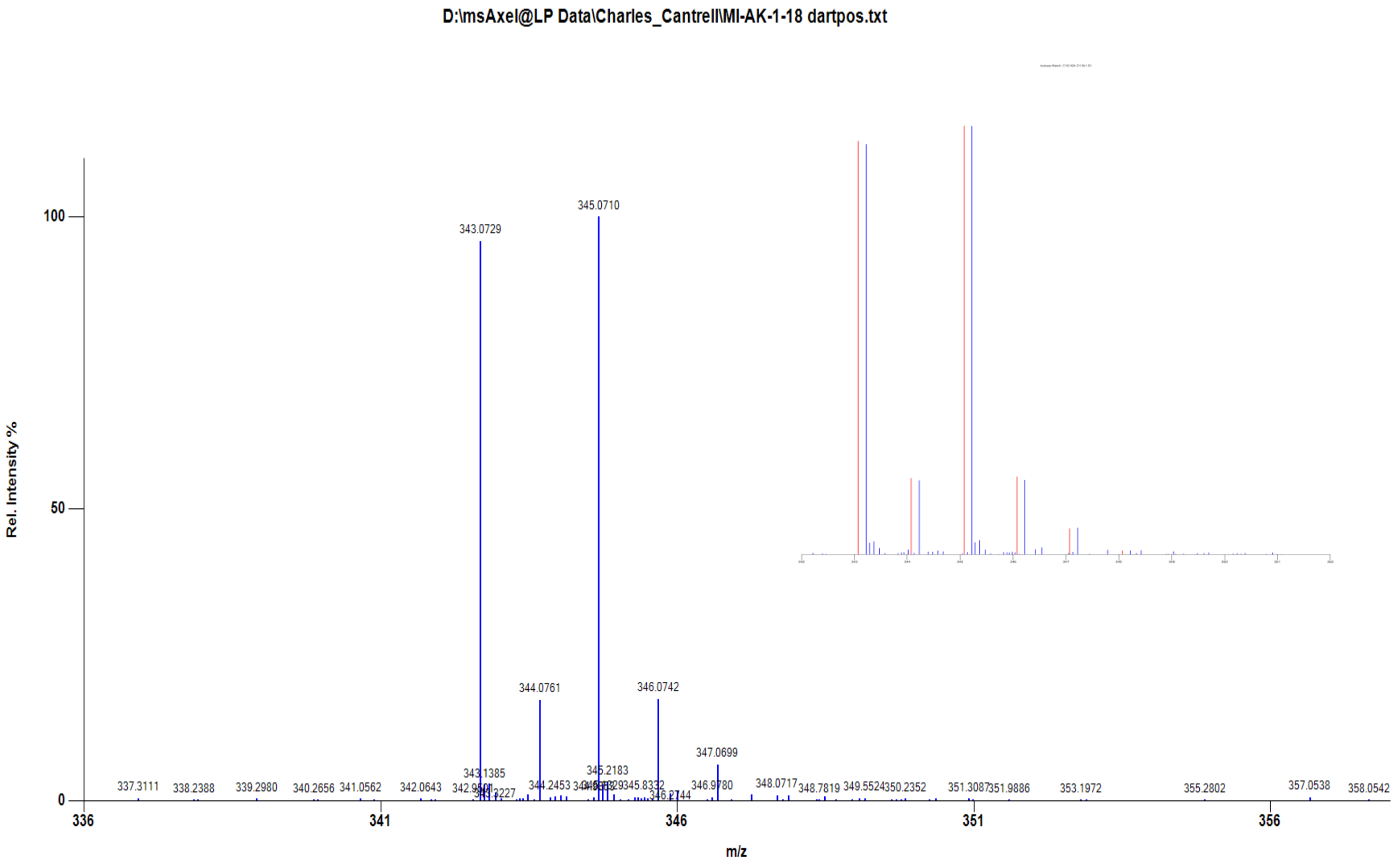

Figure S27: HRMS of **2f**
